# Discovery of a subgenotype of human coronavirus NL63 associated with severe lower respiratory tract infection in China, 2018

**DOI:** 10.1080/22221751.2020.1717999

**Published:** 2020-01-29

**Authors:** Yanqun Wang, Xin Li, Wenkuan Liu, Mian Gan, Lu Zhang, Jin Wang, Zhaoyong Zhang, Airu Zhu, Fang Li, Jing Sun, Guoxian Zhang, Zhen Zhuang, Jiaying Luo, Dehui Chen, Shuyan Qiu, Li Zhang, Duo Xu, Chris Ka Pun Mok, Fuchun Zhang, Jingxian Zhao, Rong Zhou, Jincun Zhao

**Affiliations:** a State Key Laboratory of Respiratory Disease, National Clinical Research Center for Respiratory Disease, Guangzhou Institute of Respiratory Health, the First Affiliated Hospital of Guangzhou Medical University, Guangzhou, People’s Republic of China; b Institute of Infectious disease, Guangzhou Eighth People's Hospital of Guangzhou Medical University, Guangzhou, People’s Republic of China; c The HKU–Pasteur Research Pole, School of Public Health, Li Ka Shing Faculty of Medicine, The University of Hong Kong, Hong Kong, People’s Republic of China

**Keywords:** human coronavirus NL63, new subgenotype, pneumonia, whole-genome sequencing, phylogenetic analysis, viral entry

## Abstract

Human coronavirus NL63 (HCoV-NL63) is primarily associated with common cold in children, elderly and immunocompromised individuals. Outbreaks caused by HCoV-NL63 are rare. Here we report a cluster of HCoV-NL63 cases with severe lower respiratory tract infection that arose in Guangzhou, China, in 2018. Twenty-three hospitalized children were confirmed to be HCoV-NL63 positive, and most of whom were hospitalized with severe pneumonia or acute bronchitis. Whole genomes of HCoV-NL63 were obtained using next-generation sequencing. Phylogenetic and single amino acid polymorphism analyses showed that this outbreak was associated with two subgenotypes (C3 and B) of HCoV-NL63. Half of patients were identified to be related to a new subgenotype C3. One unique amino acid mutation at I507 L in spike protein receptor binding domain (RBD) was detected, which segregated this subgenotype C3 from other known subgenotypes. Pseudotyped virus bearing the I507 L mutation in RBD showed enhanced entry into host cells as compared to the prototype virus. This study proved that HCoV-NL63 was undergoing continuous mutation and has the potential to cause severe lower respiratory disease in humans.

## Article summary line

This study reports an outbreak of severe lower respiratory illness caused by two subgenotypes (C3 and B) of human coronavirus NL63 (HCoV-NL63). The new subgenotype C3 with enhanced viral entry into host cells accounts for half of patients, which alerted that HCoV-NL63 which consist of multiple subgenotypes, is undergoing continuous mutation, and has the potential to cause large-scale severe infections in humans.

## Introduction

Six coronaviruses are known to infect humans and cause respiratory disease, including human coronavirus (HCoV) 229E [1], OC43 [2], severe acute respiratory syndrome CoV (SARS-CoV) [3], NL63 [4], HKU1 [5] and Middle East respiratory syndrome CoV (MERS-CoV) [6]. SARS-CoV and MERS-CoV are highly pathogenic coronaviruses that caused severe and fatal respiratory infections in humans. The SARS-CoV pandemic infected over 8000 people worldwide [7]. As of 9 September 2019, 2458 MERS cases with 848 deaths (34.5% mortality) were reported to World Health Organization (WHO). HCoV-229E, OC43, NL63 and HKU1 are endemic in humans and mainly cause mild respiratory infections worldwide [8,9].

HCoV-NL63 has been prevalent worldwide for many years. The majority of HCoV-NL63 infections in human are mild, although occasionally NL63 causes pneumonia or central nervous system diseases in susceptible individuals including young children, elderly and immunosuppressed patients [10,11]. HCoV-NL63 primarily infects upper respiratory tract and most of HCoV-NL63 infections are acquired during childhood. Neutralizing activity directed against HCoV-NL63 is common in sera from adults and rarely in infant’s serum [12]. During 2009 and 2016, HCoV-NL63 accounted for about 0.5% (60/11399) of all acute respiratory tract infections in hospitalized pediatric patients in Guangzhou, China [13], most of these cases associated with HCoV-NL63 were considered to be evidence of endemic infection and no outbreaks were reported.

Here, we identified a cluster of 23 hospitalized pediatric patients with severe lower respiratory tract infection caused by two subgenotypes (C3 and B) of HCoV-NL63, and half of the patients were caused by a new subgenotype C3 which was first reported here. A unique mutation (I507 L) in receptor-binding domain (RBD) was detected in the new subgenotype of HCoV-NL63 associated with increased viral entry into host cells indicating that HCoV-NL63 was undergoing continuous mutation which potentially could enhance HCoV-NL63 virulence and promote transmission. This study showed that HCoV-NL63 had the potential to cause epidemics in humans and it may be a more important human pathogen than is commonly believed. Efforts should be paid to monitor genetic changes in HCoV-NL63 genome and also its pathogenicity and prevalence in the human population [14].

## Materials and methods

### Clinical specimens

Respiratory samples, including nasopharyngeal aspirates or induced sputum, were collected for viral infection diagnosis from hospitalized pediatric patients by medical professionals at The First Affiliated Hospital of Guangzhou Medical University in 2018 and stored at −80°C until testing. Children with obvious respiratory symptoms (fever, cough, etc.) were included in this study, however, the patients with evidence of injuries, COPD, immune diseases, chronic diseases, metabolic disorders were excluded from this study. This study was performed in strict accordance with human subject protection guidance proved by the Research Ethics Committee of The First Affiliated Hospital of Guangzhou Medical University.

### Screening of HCoV-NL63 positive samples

All the respiratory samples were first screened for HCoV-NL63 infection using TaqMan real-time PCR assays (Guangzhou HuYanSuo Medical Technology Co., Ltd.) [13,15]. HCoV-NL63 positive samples were simultaneously tested for 14 other common respiratory pathogens, including HCoV-229E, HCoV-OC43, HCoV-HKU1, influenza A virus (Flu A), influenza B virus (Flu B), enterovirus (EV), human bocavirus (HBoV), human rhinovirus (HRV), respiratory syncytial virus (RSV), adenovirus (ADV), four types of human parainfluenza virus (HPIV), Mycoplasma pneumoniae (MP), human metapneumovirus (HMPV) and Chlamydia pneumonia (CP). Statistical analysis was performed on the proportion of single- and co-infection.

### Complete genome sequencing

Viral nucleic acid was extracted from HCoV-NL63 positive respiratory samples using a QIAamp viral RNA extraction kit (QIAGEN, Hilden) following the manufacturer's instructions and was used for sequence-independent single-primer amplification (SISPA) as previously described [16]. Purified DNA was sent for library preparation and sequencing using Illumina Hiseq 2500 instrument (BerryGenomics Company, Beijing). Data analysis was performed using CLC Genomics Workbench version 11. Paired-end reads (2 × 150 bp) were assembled into contigs and aligned to viral metagenomic database comprising all viral reference sequences (all viral reference sequences in NCBI: 12,209 strains). In parallel, sets of specific primer pairs were designed and used to amplify the complete genome of HCoV-NL63. The complete genomes of HCoV-NL63 were annotated based on the annotation of the HCoV-NL63 prototype strain (NC_005831.2).

### Phylogenetic and single amino acid polymorphism analysis

All available HCoV-NL63 complete genomes (53 strains) well collected from GenBank up to date. PhyML software [17] was used for phylogenetic analyses based on complete genome, orf1ab and spike gene sequences, respectively. HCoV-NL63 strains were divided into three genotypes (genotypes A, B and C) based on phylogenetic analyses of partial S genes. Genotype A was further divided into subgenotypes A1, A2 and A3. Genotype C was divided into three subgenotypes C1, C2 and C3. Neighbour-Joining and Maximum likelihood methods derived from MEGA 7.0 were also used to confirm the topological structure. The nucleic acid sequence alignment was performed using MAFFT software [18]. Corresponding spike gene sequences were collected and were used to performed amino acid alignment using MEGA 7 software [19]. Single amino acid residues were extracted for comparison analysis between different subgenotypes.

### Genetic recombination analyses

Bootscanning analyses were performed using the default settings of SimPlot software [20]. ChinaGD02 strain was used as the query sequence and was compared to those of other HCoV-NL63 subgenotype strains.

### Estimated divergence time of the new HCoV-NL63 circulating in Guangzhou

To understand the temporal constraints on the origin of HCoV-NL63 subgenotypes, BEAST software was used to estimate the most recent common ancestor (tMRCA) of the new subgenotype circulating in Guangzhou, based on nucleotide sequences of spike gene. Analyses were conducted under the best-fit nucleotide substitution model (GTR  +  I + G) and using a relaxed (uncorrelated lognormal) molecular clock model. The Markov chain Monte Carlo (MCMC) analysis was performed with 1 × 10^8^ generations and was sampled every 1000 generations. Convergence was assessed using Tracer software, based on the effective sampling size after a 10% burn-in.

### Pseudotyped virus production and viral entry assay

HCoV-NL63 pseudotyped viruses were generated with two methods, including lentiviral system and vesicular stomatitis virus (VSV) pseudotyping system. Lentiviral pseudotyped with HCoV-NL63 spike protein were produced as previously described [21]. Briefly plasmid encoding spike protein was co-transfected into 293 T cells with reporter plasmid plenti-GFP-luc and helper plasmid psPAX2, viral supernatant were harvested 48 h after transfection and normalized using commercial p24 enzyme-linked ELISA kit (Clontech, Japan). VSV-ΔG-luc pseudotyped with HCoV-NL63 spike was prepared using recombinant (rVSV-ΔG-luc) as described previously [22]. In short, plasmid encoding wild-type (reference strain Amsterdam I, accession number: NC_005831.1) or mutant HCoV-NL63 spike protein was transfected into 293 T cells, 24 h later transduced target cells were infected with rVSV-ΔG-luc, viral supernatant was harvested 24 h after transduction. Both VSV-ΔG-luc pseudotyped (prototype or mutant) were recovered under the same condition. Plasmid encoding mutant spike protein (I507 L) was generated using a Mut Express II Fast Mutagenesis Kit (Nanjing Vazyme Biotech, China) according to the manufacturer’s instructions. The infected Huh7 cells were lysed 48 h after infection, and the efficiency of viral entry was measured by comparing the luciferase activity between pseudotyped viruses bearing the wild-type or mutant S protein.

### Accession numbers

The complete and partial genomes of human coronavirus NL63 identified in this study were deposited in GenBank under the accession numbers: MK334043–MK334047, MK342125–MK342133.

## Results

### Clinical information and co-infections of patients infected with HCoV-NL63

**Figure 1.**
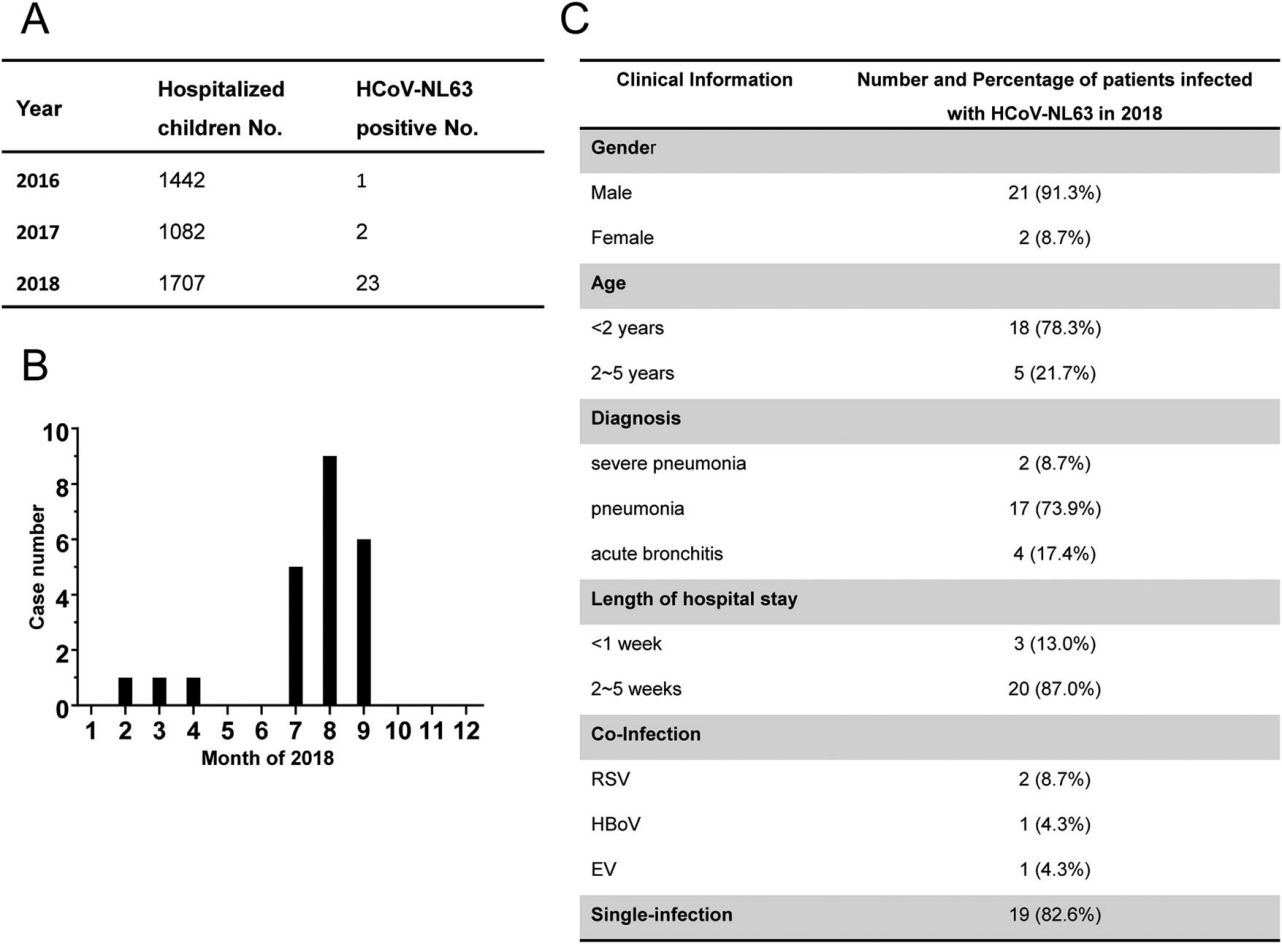
**Identification of a cluster of patients with severe lower respiratory disease caused by HCoV-NL63. (A)** Prevalence of HCoV-NL63 by year in the pediatric inpatient surveillance study at the First Affiliated Hospital of Guangzhou Medical University. **(B)** Case number of HCoV-NL63 infection by month in the pediatric inpatient surveillance study in 2018. **(C)** Clinical information of the HCoV-NL63 positive inpatients in 2018.

Between 2016 and 2017, there were only three HCoV-NL63 positive pediatric patients detected in the First Affiliated Hospital of Guangzhou Medical University. However, in 2018 there was a sharp increase of HCov-NL-63 infection. Twenty-three cases were detected in 2018, 20 of which were detected from July to September 2018 (Figure 1A and B). The clinical information of the HCoV-NL63 infected patients was summarized in Figure 1(C). The majority of patients who were infected with HCoV-NL63 had pneumonia, and two patients had severe pneumonia. Their ages ranged from 5 months to 5 years old. Clinical manifestation of these patients included cough, fever, coryza, shortness of breath, wheezing and abnormal pulmonary breath sound. Samples from HCoV-NL63 positive patients were also tested for other 14 common respiratory pathogens. Of the 23 patients, 19 (82.6%) of them were infected only with HCoV-NL63, while 4 (17.4%) patients were found to be co-infected with other pathogens, such as RSV, HBoV and EV. These data clearly showed that most of the patients with lower respiratory tract infection (LRTI) were caused by HCoV-NL63 but not co-infected with other pathogens. HCoV-NL63 infection presented here preferentially caused severe lower respiratory tract infection rather than upper respiratory tract infection. No evidence of injuries, COPD, immune diseases, chronic diseases, metabolic disorders et al were detected in these patients. Comparison of clinical characteristics including age, co-infection and clinical symptoms showed no obvious differences between different subgenotypes infections (Table S1). Considering the increasing case number and the proportion of HCoV-NL63 single infection, this should be a small-scale outbreak associated with HCoV-NL63 in Guangzhou, China, in 2018 indicating HCoV-NL63 is a more important human pathogen than is commonly believed.

### Phylogenetic analysis revealed a new subgenotype of HCoV-NL63 virus circulating in South China

**Figure 2.**
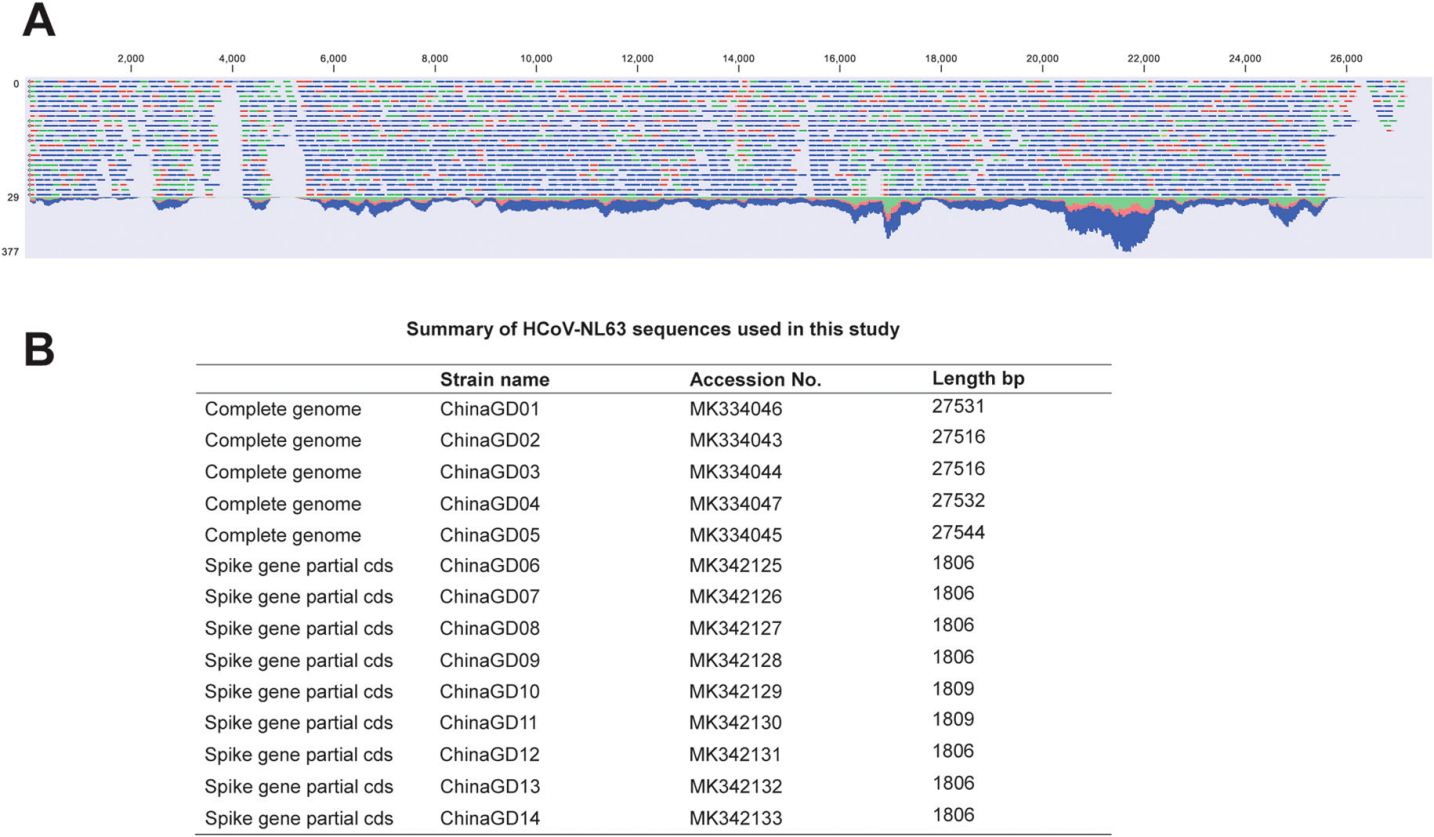
**Whole-genome sequencing of HCoV-NL63 identified in this study.** (**A**) Whole genome coverage of HCoV-NL63 performed by CLC Genomics Workbench software version 11. (**B**) Summary of HCoV-NL63 sequences obtained in this study.

**Figure 3.**
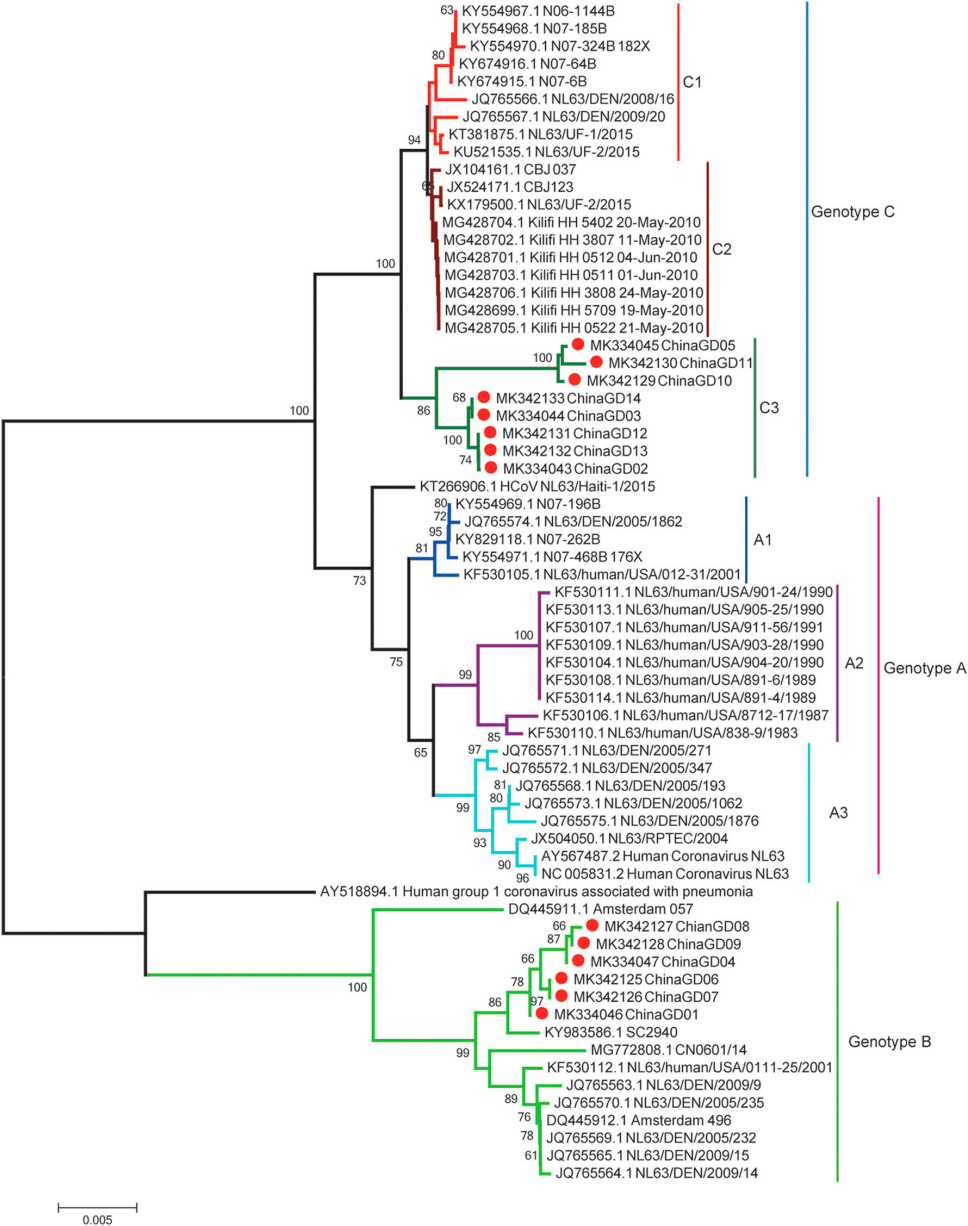
**Phylogenetic analyses based on partial spike genes of HCoV-NL63 identified in this study.** Fourteen HCoV-NL63 spike gene partial sequences detected in this study were used for phylogenetic analysis by MEGA 7.0 software using Neighbour-joining method and further confirmed the presence of new subgenotype of HCoV-NL63. Bootstrap values greater than 60% were considered statistically significant for grouping.

**Figure 4.**
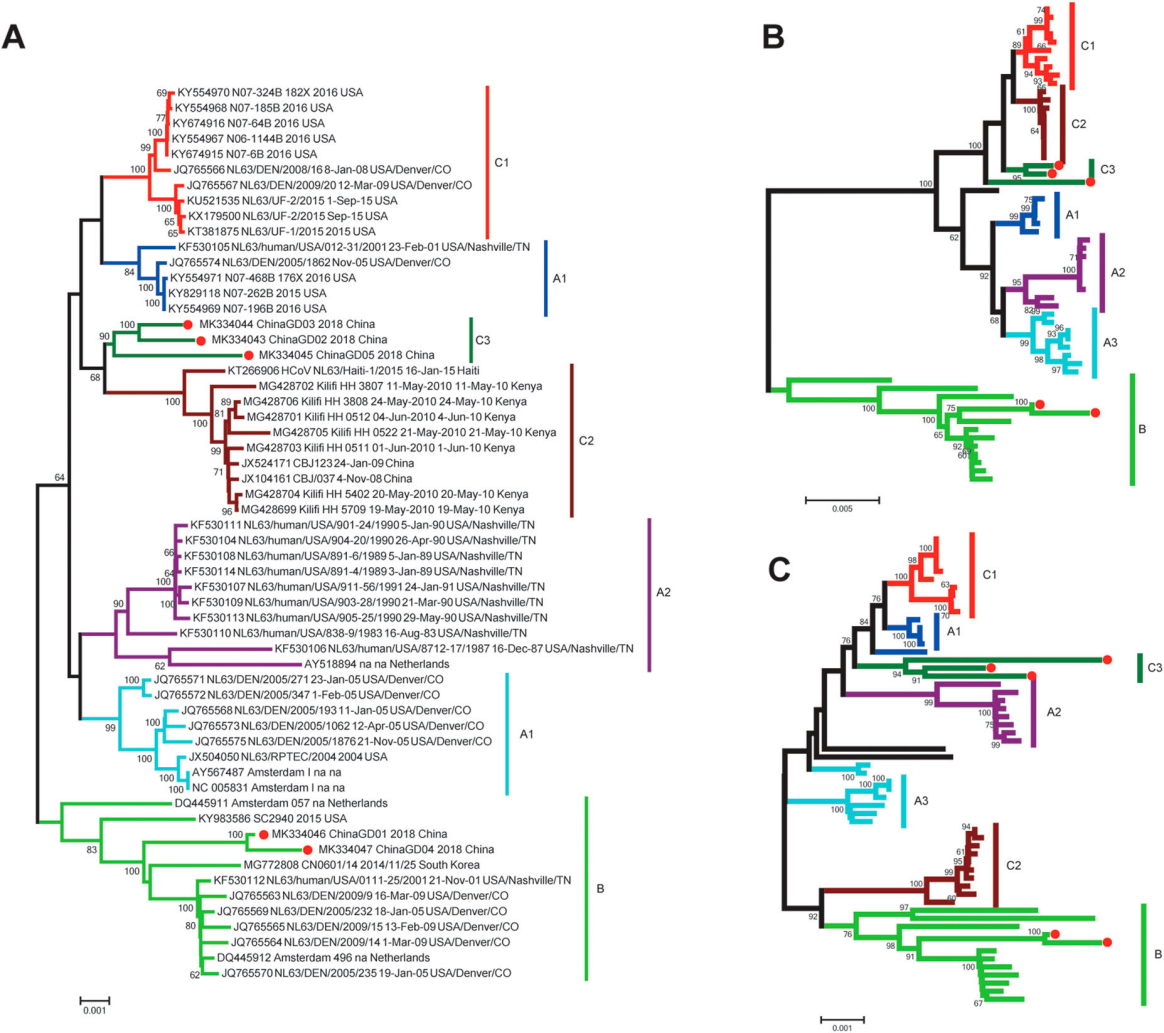
**Phylogenetic analysis based on complete genome, S and ORF1ab genes of HCoV-NL63.** All available HCoV-NL63 complete genomes (53 strains) from GenBank were collected and used for the evolutional analysis using MEGA 7.0 with 1000 bootstrap replications, Bootstrap values greater than 60% were considered statistically significant for grouping. (**A**). Five complete genomes derived from this study were in red. Nucleotide sequence alignments were created using MAFFT. Corresponding spike (**B**) and orf1ab (**C**) genes were used for the genotype identification.

HCoV-NL63 positive respiratory samples were collected and used for next-generation sequencing. Clean data was used for assembling or mapping against the reference sequence (NC_005831.2) of HCoV-NL63 (Figure 2A). Using both next-generation and Sanger sequencing technology, five complete genomes and nine partial spike protein sequences of HCoV-NL63 were obtained and analysed in this study (Figure 2B). Other HCoV-NL63 sequences were not obtained because of low viral load in respiratory specimens. To investigate genetic relationship between HCoV-NL63 strains detected in this study and other HCoV-NL63 strains available in GenBank, we performed phylogenetic analyses based on partial spike gene sequences. All available HCoV-NL63 strains were collected and divided into three genotypes (genotypes A, B and C) based on phylogenetic analyses of partial S genes (Figure 3). Genotype A was further divided into subtypes A1, A2 and A3. Genotype C contained three subgenotypes C1, C2 and C3. We also performed phylogenetic analyses based on complete genome, orf1ab and spike genes, respectively (Figure 4). Phylogenetic trees based on orf1ab, spike genes and complete genome showed similar topologies with seven sub-genotypes, which further confirmed the phylogenetic structure and supported a new subgenotype C3 circulating in Guangzhou, China (Figure 4). There were two subgenotypes (C3 and B) of HCoV-NL63 circulating in South China, Guangzhou, which associated with increasing numbers of severe respiratory illness in 2018. Significantly, the ChinaGD02, ChinaGD03, ChinaGD05, ChinaGD10 strains presented in this study shared a common origin and formed a new subgenotype C3 on the same branch with other HCoV-NL63 subgenotypes. Meanwhile the old genotype B was also circulating in China which was identified previously from American clinical specimen [23]. In summary, the discovery of the new subgenotype C3 emphasized the risk of an epidemic caused by a mutated HCoV-NL63.

### Recombination analysis and estimated divergence time of the new subgenotype of HCoV-NL63

**Figure 5.**
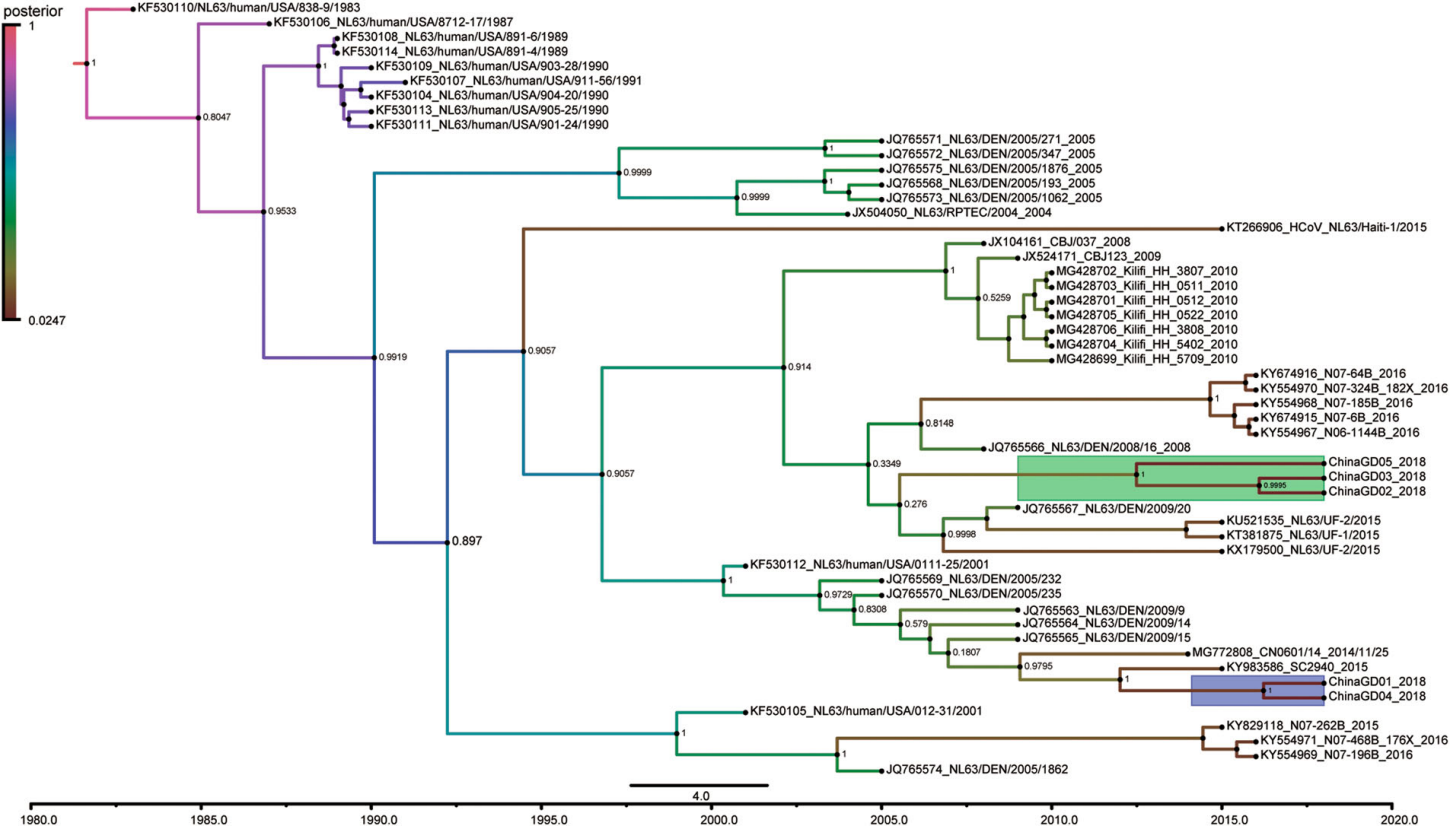
**Time-resolved phylogenetic analysis of spike gene of HCoV-NL63 strains.** The year of sampling, strain name and accession number are on the tip labels. Node labels indicate the posterior probabilities. Strains of subgenotypes C3 and B detected in Guangzhou were indicated in green and purple. BEAST software was used to estimate the most recent common ancestor (tMRCA) of the new subgenotype circulating in Guangzhou, based on nucleotide sequences of spike gene. Analyses were conducted under the best-fit nucleotide substitution model (GTR  +  I + G) and using a relaxed (uncorrelated lognormal) molecular clock model.

The phylogenetic analysis of the corresponding orf1ab and spike gene sequences generated similar topologies about subgenotype C3. No obvious recombination events were detected in subgenotype C3 using Simplot software analysis (Figure S1). To date the divergence time of the new subgenotype of HCoV-NL63 detected in Guangzhou, the time to most recent common ancestor for the newHCoV-NL63 was estimated to be between 5 and 6 years before the identification in 2012–2013 (Figure 5). Bayesian skyline plot analyses were estimated to depict the relative genetic diversity of HCoV-NL63 in spike gene over time (Figure S2), HCoV-NL63 showed moderate increase in genetic diversity between 1989 and 2015, while showed slight decrease in 2018 due to the limited number of update HCoV-NL63 sequences. Given the phylogenetic and recombination analysis, we concluded that the new subgenotype C3 of HCoV-NL63 was derived from genetic mutation recently.

### I507l mutation in RBD promoted viral entry

**Figure 6.**
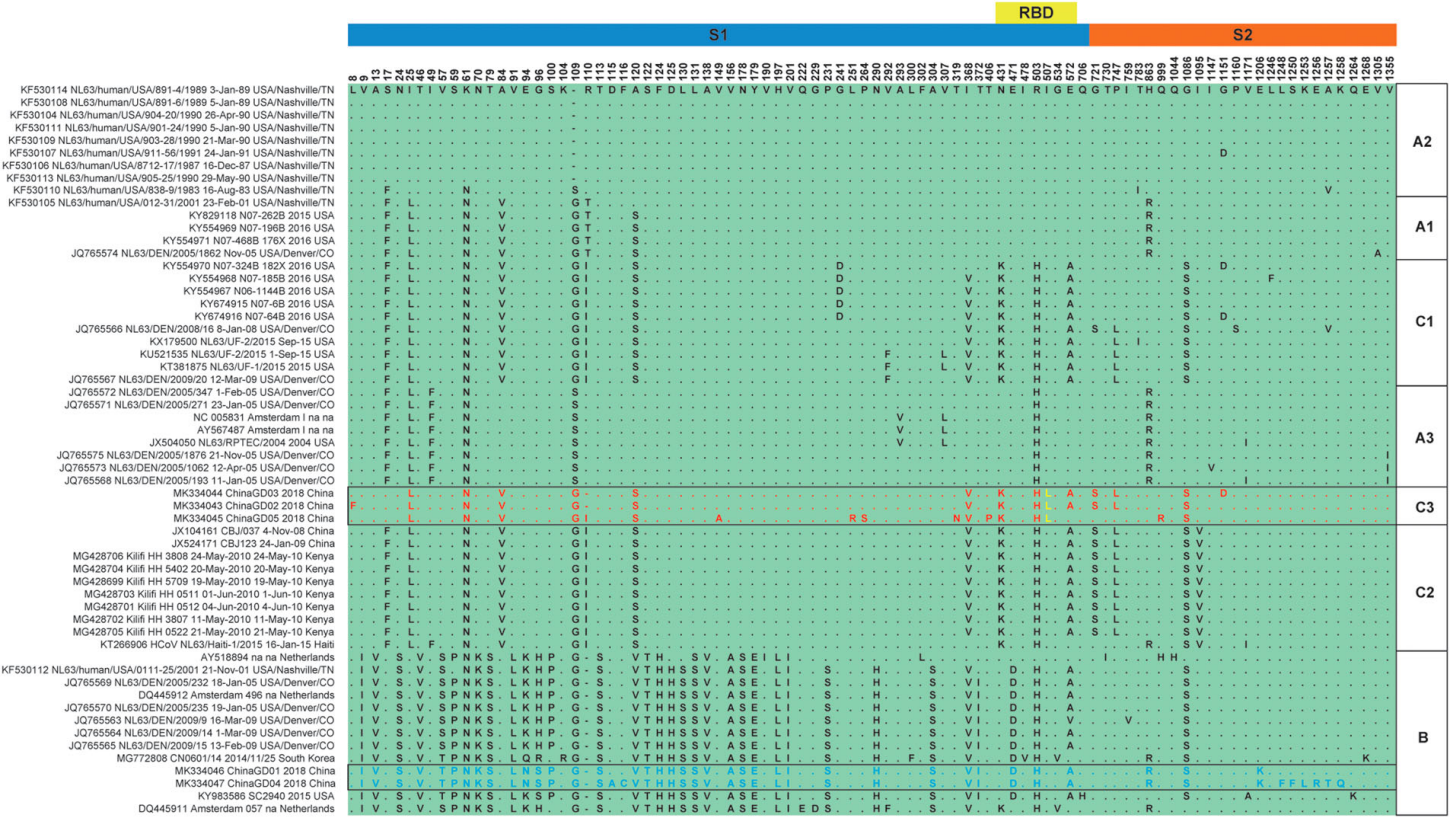
**Single amino acid polymorphism analysis of HCoV-NL63 spike protein.** All available HCoV-NL63 complete genomes were aligned, and corresponding spike proteins were retrieved and used for single amino acid polymorphism analysis. Most of SAP lies in S1 domain, and one unique mutation I507L was identified in RBD of spike in yellow.

The spike protein plays a key role in coronaviruses’ entry and pathogenesis. To further characterize the HCoV-NL63 strains detected in this study, corresponding spike protein sequences of HCoV-NL63 strains were extracted and used for single amino acid polymorphism analysis (SAP). As shown in Figure 6, there were 89 single amino acid polymorphism sites recognized in the spike proteins. The subgenotype-specific SAP in spike protein further confirmed the rationality of the new subgenotypic classification (subgenotype A1, A2, A3, B, C1, C2, and C3). Residues from subgenotype C3 and genotype B strains are shown in red and blue, respectively. The receptor binding domain (RBD) resides in the central portion of spike protein, residues 476–616 [24,25]. Of note, one signature amino acid mutation at 507 L of the spike receptor binding domain (RBD) was detected in all strains of subgenotype C3 which segregated from other different subgenotypes. In addition, amino acid alignment analysis of HCoV-NL63 partial spike protein indicated that most of the HCoV-NL63 detected here had the special mutation I507 L which belongs to subgenotype C3 (Figure S3). This mutation may be associated with change in virulence or transmission of HCoV-NL63 in humans.

**Figure 7.**
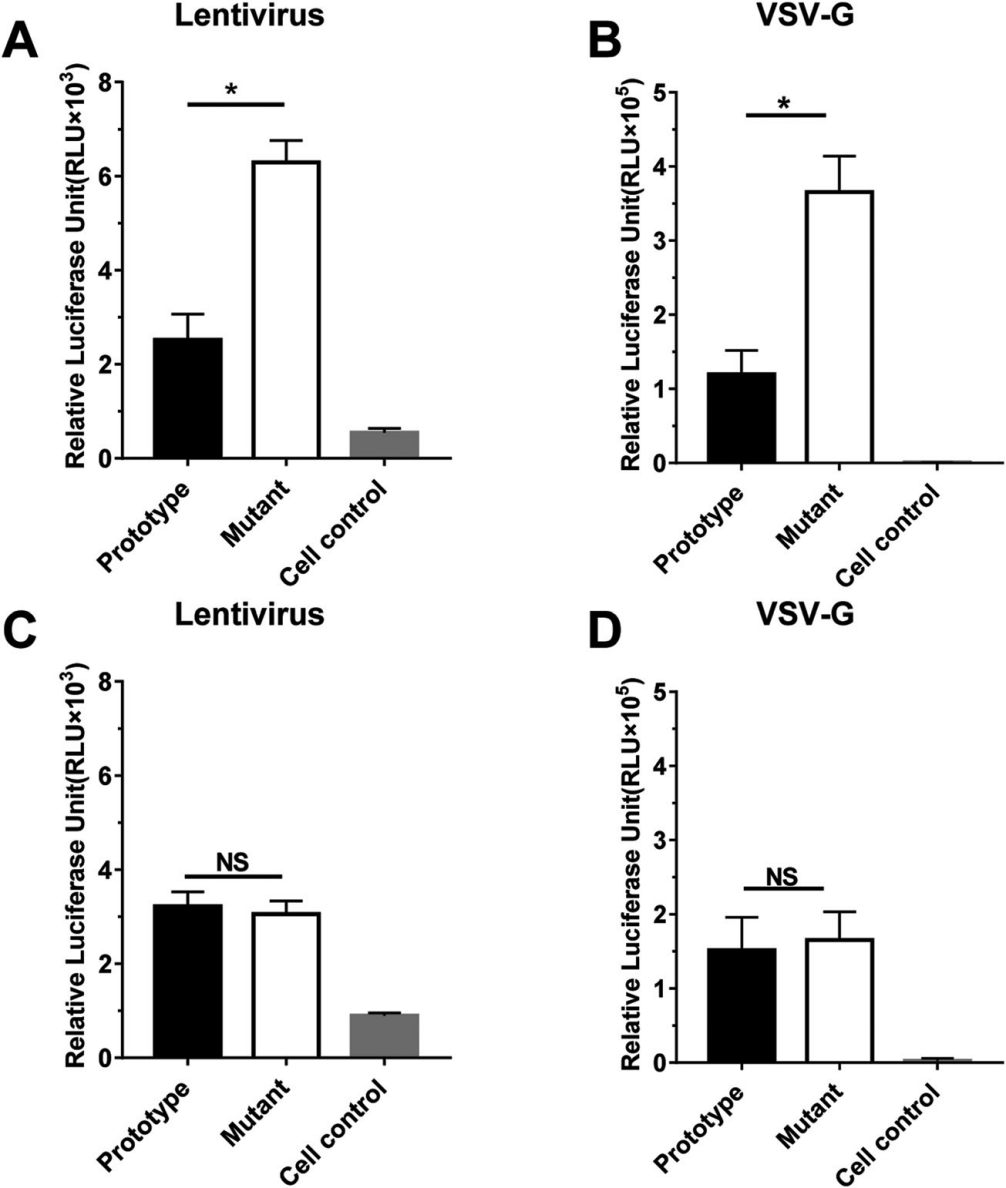
**I507L mutation in RBD promoted viral entry.** The efficiency of viral entry was analysed using HCoV-NL63-S pseudotyped viruses bearing the wild-type (WT) or I507L (**A**, **B**) or E471D (**C**, **D**) mutant S proteins in Huh7 cells. Cell control serves as the negative control to determine background. Two different viral pseudotypes were employed in this study, including lentivirus system (**A, C**) and VSV system (**B, D**). Huh7 cells were infected with indicated pseudotyped viruses in triplicates. Luciferase activities were measured 72 h post infection. A Student's *t* test was used to analyse differences in mean values between groups. *P* values of <0.05 were considered statistically significant. Data are representative of three independent experiments.

To investigate the biological relevance of I507 L mutation in spike RBD, pseudotyped HCoV-NL63 were generated bearing WT (reference strain Amsterdam I, accession number: NC_005831.1) and I507 L mutant S protein. The infectivity of pseudotyped HCoV-NL63 was detected by luciferase reaction in Huh7 cells. Cell control serves as the negative control to determine background. As showed in Figure 7(A and B), in both lentiviral and VSV system mutant pseudotyped (507 L) showed increased viral entry into susceptible cells compared to WT. The higher viral entry may be associated with increased transmission and case number in human in South China. Sequences alignment between subgenotype C3 and B indicated only one SNP (E471D) was located in RBD region. To directly compare viral entry between subgenotypes B and C3, pseudotype bearing E471D mutation was prepared. No difference was observed between E471D mutant and WT in Figure 7(C and D). The crystal structure of HCoV-NL63 RBD complexed with its receptor ACE2 has been published previously [24] and three key RBMs (receptor binding motifs) were defined as RBM1 (residues 493–513), RBM2 (residues 531–541) and RBM3 (residues 585–590). The mutation I507 L lied in RBM1 and may promote viral entry during infection. More epidemiological surveys should be performed. Discovery of the new subgenotype emphasized the risk of increased pathogenicity of HCoV-NL63 as evolution.

## Discussion

It has been well documented that SARS-CoV and HCoV-NL63 share the same receptor, angiotensin converting enzyme (ACE)-2, for entry into the host cells [26]. However, the consequences following viral entry are quite different. SARS-CoV caused severe respiratory distress, while HCoV-NL63 showed much lower affinity with ACE2 molecule and led to moderate respiratory infections, which were restricted to the upper respiratory tract. Outbreak of severe respiratory infections among children caused by HCoV-NL63 is rarely reported [27,28]. In China, HCoVs (OC43, NL63, HKU1 and 229E) are responsible to about 4.3% of hospitalized children with acute respiratory tract infection [13], and HCoV-OC43 was the predominant circulating HCoV. Little is known about the genetic variability of HCoV-NL63 in China and worldwide since only a few complete genomes are available [10,15,29].

Here, we identified a cluster of 23 hospitalized pediatric patients with severe lower respiratory infection who were infected with HCoV-NL63 in Guangzhou, 2018. Most of these patients showed symptoms of pneumonia or severe pneumonia. This represented a significant increase in case numbers of HCoV-NL63 infection as compared to last 10 years in the corresponding period of time indicating a small-scale outbreak of severe respiratory illness caused by HCoV-NL63. In addition, phylogenetic and recombination analyses demonstrated that two subgenotypes (C3 and B) of HCoV-NL63 were associated with severe lower respiratory tract disease and a new subgenotype was first discovered and showed different evolutionary trends. Previous reports showed that HCoV-NL63 infections in China are distributed across the whole year without an obvious seasonal distribution or presence of clusters in China [30,31]. The epidemic characteristics benefited viral continuous transmission and fast evolution.

HCoV-NL63 mainly caused mild upper respiratory tract infectious symptoms; fewer of them progressed to lower respiratory tract infection (LRTI) and pneumonia. The new subgenotype circulating in Guangzhou primarily caused LRTI in hospitalized children and showed the sign of fast transmission. Based on previous reports, HCoV-NL63 was mainly divided into three genotypes, including A, B and C. However, as the continuous identification of HCoV-NL63 related sequences, the genotyping classification cannot meet the requirement for sequence analysis, especially for discovery of new subgenotype. Here, we divided all the HCoV-NL63 available into seven subgenotypes (subgenotypes A1, A2, A3, B, C1, C2 and C3). The subgenotype-specific SAP analysis of spike protein also supported this classification. Comparison of clinical characteristics between subgenotype C3 and B infected patients showed no significant difference. More genotype-specific cases were required to explore the relationship between clinical symptoms and genotype or co-infection.

Only two highly pathogenic human coronaviruses, SARS-CoV and MERS-CoV, have been identified thus far. Due to various reasons (geographical restriction, inefficient human to human transmission route, etc.) their ability to spread efficiently enough to cause a global pandemic is limited [32,33]. HCoV-NL63 has been prevalent worldwide for about 1000 years [14], although most of the infections associated with HCoV-NL63 are mild. As the continuous transmission and evolution, the change of pathogenicity is inevitable. Here, the new subgenotype of HCoV-NL63 causing LRI appeared to be far more infectious due to the unique mutation I507L in RBD. Considering HCoV-NL63 and SARS-CoV share the same cellular receptor, a better understanding of pathogenicity and epidemiology of newHCoV-NL63 subgenotype is of particular importance in view of potential enhanced virulence or transmission. Epidemiological and phylogenetic studies are required periodically to evaluate the risk of an epidemic caused by newHCoV-NL63.

## Supplementary Material

Supplemental Material
