# Supplementary material for: Discovery of a subgenotype of human coronavirus NL63 associated with severe lower respiratory tract infection in China, 2018
Source: Emerg Microbes Infect. 2020 Jan 29;9(1):246–55. doi: 10.1080/22221751.2020.1717999 (PMC7034077; doi:10.1080/22221751.2020.1717999)
Supplement: Supplemental Material [file TEMI_A_1717999_SM9841.zip › supplementary materials/Table S1.docx]

**Table S1. Clinical characteristics of HCoV-NL63 detected in this study**

| NL63 strain | Genotype | Sample date(month/year) | Sex | Co-infection | Admitted | Symptoms | Discharge diagnosis | Age (year) |
| --- | --- | --- | --- | --- | --- | --- | --- | --- |
| ChinaGD01 | B | 7/2018 | M |  | Y | headache,fever,cough | Pneumonia | 1 |
| ChinaGD02 | C3 | 9/2018 | M | RSV | Y | headache,fever | Pneumonia | 2 |
| ChinaGD03 | C3 | 8/2018 | M |  | Y | fever, shortness of breath | severe pneumonia | 3 |
| ChinaGD04 | B | 9/2018 | F |  | Y | cough | acute bronchitis | 1 |
| ChinaGD05 | C3 | 9/2018 | M | HBoV | Y | cough | pneumonia | 1 |
| ChinaGD06 | B | 8/2018 | M |  | Y | headache,fever | pneumonia | 4 |
| ChinaGD07 | B | 8/2018 | M | RSV | Y | cough | acute bronchitis | 2 |
| ChinaGD08 | B | 7/2018 | M |  | Y | headache,fever | pneumonia | 1 |
| ChinaGD09 | B | 4/2018 | M |  | Y | fever, shortness of breath | severe pneumonia | 2 |
| ChinaGD10 | C3 | 7/2018 | F |  | Y | cough | pneumonia | 2 |
| ChinaGD11 | C3 | 9/2018 | M |  | Y | headache,fever | pneumonia | 2 |
| ChinaGD12 | C3 | 8/2018 | M |  | Y | headache,fever | pneumonia | 2 |
| ChinaGD13 | C3 | 9/2018 | M |  | Y | headache,fever | pneumonia | 2 |
| ChinaGD14 | C3 | 3/2018 | M |  | Y | headache,fever | pneumonia | 2 |
| ChinaGD15 | C3 | 8/2018 | M |  | Y | cough | acute bronchitis | 3 |
| ChinaGD16 | C3 | 7/2018 | M | EV | Y | cough | pneumonia | 1 |
| ChinaGD17 | C3 | 8/2018 | M |  | Y | headache,fever | pneumonia | 2 |
| ChinaGD18 | unknown | 2/2018 | M |  | Y | headache,fever | pneumonia | 5 |
| ChinaGD19 | unknown | 8/2018 | M |  | Y | headache,fever | pneumonia | 2 |
| ChinaGD20 | unknown | 7/2018 | M |  | Y | headache,fever,cough | pneumonia | 2 |
| ChinaGD21 | unknown | 9/2018 | M |  | Y | headache,fever,cough | pneumonia | 3 |
| ChinaGD22 | unknown | 8/2018 | M |  | Y | fever | acute bronchitis | 1 |
| ChinaGD23 | unknown | 8/2018 | M |  | Y | fever,cough | pneumonia | 1 |

M male, F female, Y yes
