# Supplementary figures and images for: Discovery of a subgenotype of human coronavirus NL63 associated with severe lower respiratory tract infection in China, 2018
Source: Emerg Microbes Infect. 2020 Jan 29;9(1):246–55. doi: 10.1080/22221751.2020.1717999 (PMC7034077; doi:10.1080/22221751.2020.1717999)

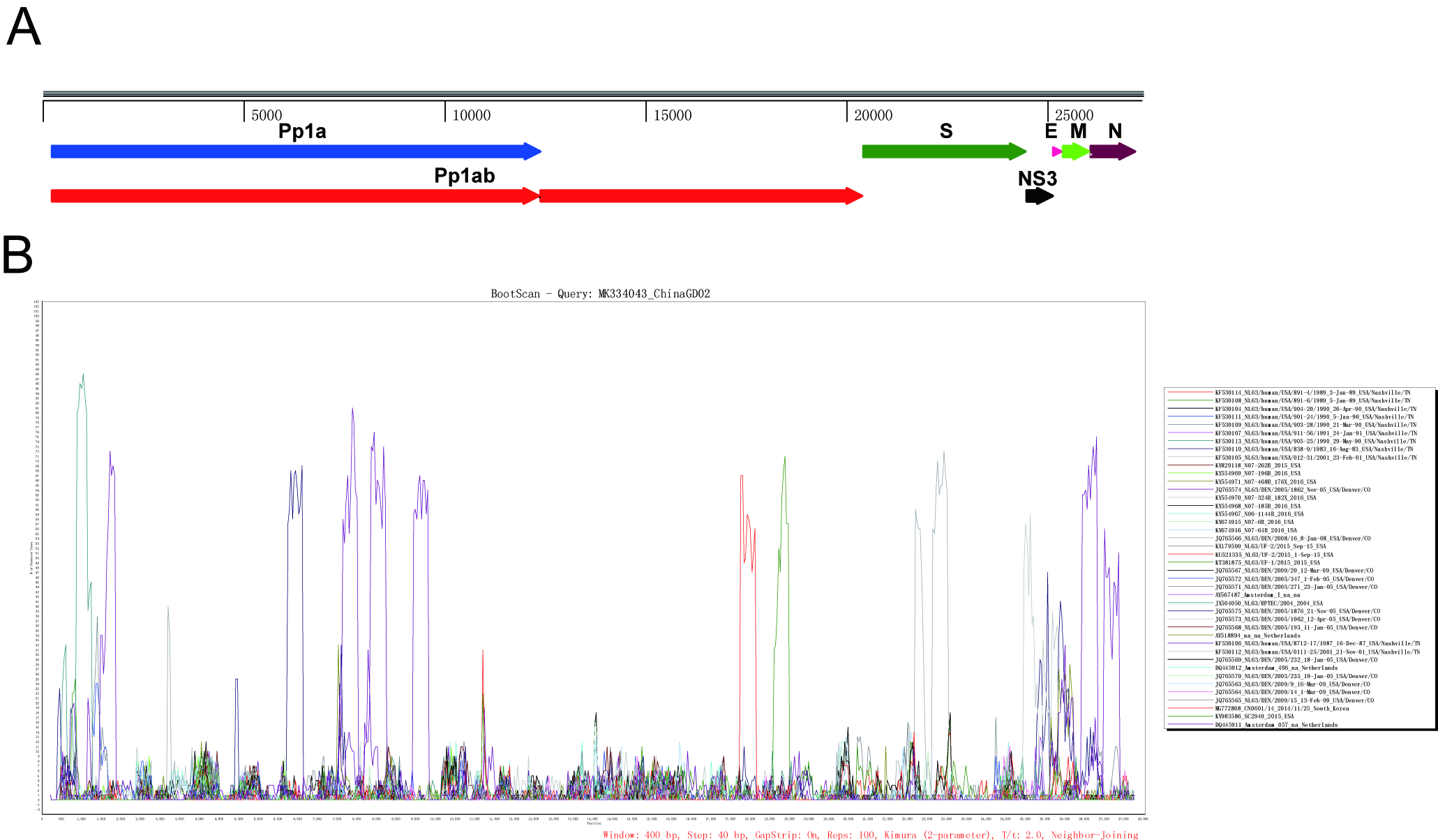

Supplement: Supplemental Material [file TEMI_A_1717999_SM9841.zip › supplementary materials/figure S1.tif]

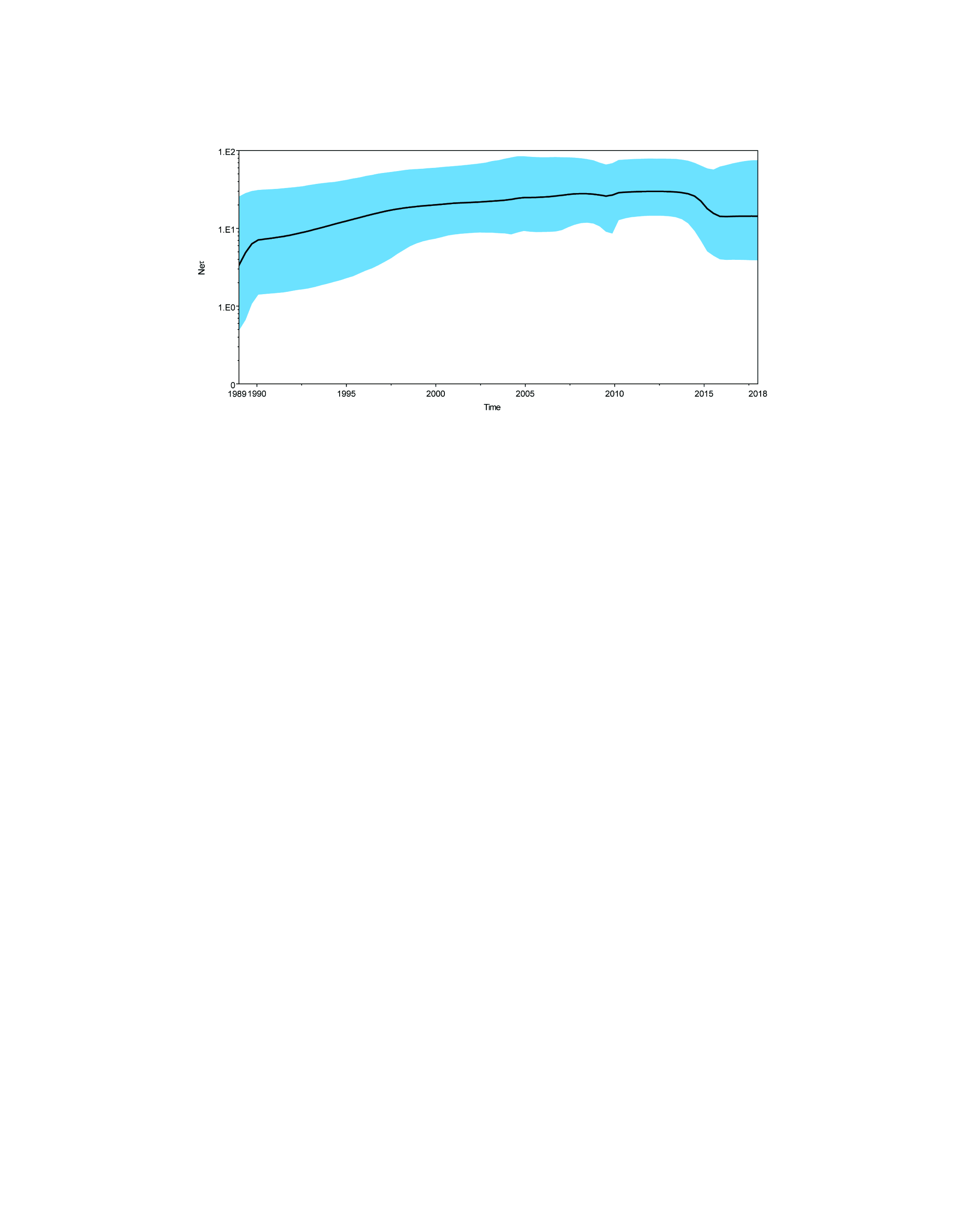

Supplement: Supplemental Material [file TEMI_A_1717999_SM9841.zip › supplementary materials/figure S2.tif]

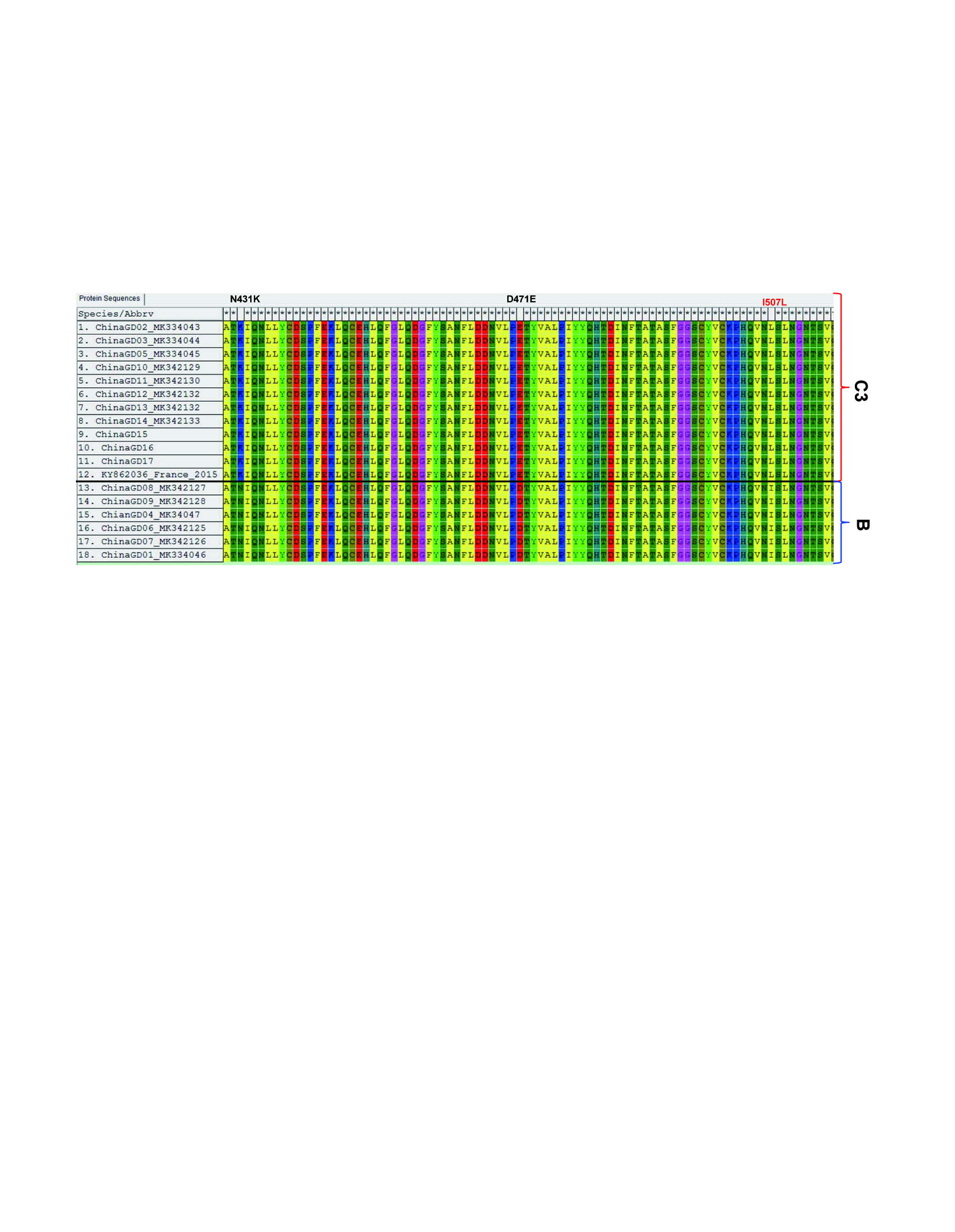

Supplement: Supplemental Material [file TEMI_A_1717999_SM9841.zip › supplementary materials/figure S3.tif]
